# Coffee; Its Use and Abuse

**Published:** 1891-07

**Authors:** I. N. Love

**Affiliations:** St. Louis, Mo.


### COFFEE; ITS USE AND ABUSE.

EXTRACT OF AN ESSAY BY DR. I. N. LOVE, ST. LOUIS, MO.

I venture opinion that there is no beverage on earth to-day which, used in moderation, expresses more comfort, contentment and calmness to the cerebral centres than coffee. But in excess, it is undoubtedly most dangerous. I doubt if the victims of alcoholic excess are as numerous as those who over indulge in coffee. Alcohol has two advantages—in that it is a food and that its excessive use is not respectable. The infusion of coffee in proper quantities aids digestion, and is a safe cerebro-spinal stimulant which is not followed by perceptible reaction.

Liebig drew attention first to the fact that this beverage contains the elements which stimulate the flow of bile. It is a decided laxative, a pronounced diuretic. The fact that the coffee belt of the world is also the "bilious belt," and the malarial belt as well as the field where noxious germs and suppurative processes most abound, is evidence of the fitness of things. No one knows better than the citizens of the hot regions of the world the value of coffee to open up the secretions which have been checked by excessive heat or the malarial influence. They know well and have known for centuries, that which has recently been receiving much attention by the medical world, particularly in Germany, viz.: the antiseptic properties of coffee.

Carl Luderitz, in the *Zeitschrift fur Hygiene*, has recently presented an interesting report concerning the influence of infusions of coffee on micro-organisms, in which he determined the influence of coffee infusions of different strength (varying from 10 to 30 per cent.) upon the growth of various forms of pathogenic and non-pathogenic micro-organisms. The coffee used in these experiments was roasted Java, and the infusions were made by adding from 10 to 30 parts of coffee by weight to 70 or 90 parts of boiling hot water. The coffee freshly roasted and ground fine, was covered with boiling water, and the infusion thus prepared was placed in a closed flask in a water bath for about ten minutes, and was then filtered through a sterilized filter. This infusion was used for making gelatin and in part directly. Where nutrient gelatin was made with this as a menstruum, it was inoculated with various forms of fungi and other micro-organisms to determine the possibility of their growth in such a medium. In other cases the organisms were added directly to the infusions of various strength, and after different periods of time inoculations were made from the infusions into other nutrient media. Luderitz found that the forms of fungi experimented with, showed more or less growth in the coffee gelatin, but the abundance of growth was in many cases distinctly less than in other media. The other micro organisms he used for his experiments were the staphylococcus pyogenes aureus,the streptococcus erysipelatosus,the typhoid bacillus, the spirillum of cholera asiatica, the bacillus anthracis, the bacillus prodigiosus, and the proteus vulgaris. All these forms of micro-organisms were greatly influenced in their life and growth by the exposure to the infusion of coffee, but some were far more susceptible than others. The bacillus prodigiosus was totally destroyed only after exposure in a 10 per cent. infusion for four days, or in a 30 per cent. infusion for one day. The typhoid bacillus was completely destroyed after exposure in a 5 per cent. infusion for three days, in a 10 per cent. infusion for from one to three days, or in a 30 per cent. infusion for one or two days. The proteus vulgaris was killed after an exposure for four days in a 10 per cent. infusion. The staphylococcus pyogenes aureus was destroyed only after an exposure for six days in a 10 per cent. infusion and for three days in a 30 per cent. infusion. The bacillus of Asiatic cholera was destroyed in a 1 per cent. infusion after 7 hours' exposure, in a 5 per cent. infusion after four hours, and in a 30 per cent. infusion after two hours. The streptococcus erysipelatosus was destroyed after an exposure of one day in a 10 per cent. infusion. The bacillus anthracis was destroyed in a 10 per cent. infusion after three hours, and in a 30 per cent. infusion after two hours.

The fact that coffee blunts sensation and increases secretion, would suggest that we educate the laity in the direction of at once giving the victims of accident a good cup of coffee, rather than the usual over stiff toddy, which, in many cases, given in excess, places the individual not only in an unfavorable condition physically, but also renders him liable to the charge from those not familiar with the facts, of having been injured on account of drunkenness.

The custom which prevails in New Orleans and generally throughout the South, of taking a cup of strong black coffee in the early morning, is an intelligent one. The people of those malarial regions have long since demonstrated the fact that the custom referred to is of great advantage as a prophylactic. The individual experience of the writer for five or six years past is strongly in favor of taking a cup of black coffee without sugar or cream, sandwiched in between two glasses of hot water before rising every morning at least one hour before breakfast. The various secretions are stimulated ; the nervous force is aroused ; an hour later a hearty meal is enjoyed and the day labor is commenced favorably, no matter how the duties of the day and night preceding may have drawn upon the physique. Another cup of coffee at four in the afternoon is sufficient to keep the energies unflagged for many hours thereafter. Taken in this manner the full effect is secured ; the stimulant devotes itself strictly to business, none of it is lost, and if the proper diet be taken at the proper times between (and the ideal diet for those who make large drafts upon their nervous systems and expect to have them honored, is hot milk), and if the above regimen be followed and accompanied by at least eight hours of sleep out of the twenty-four, the capacity for work is almost unlimited.

How many who are the victims of disease are ever consulted as to whether they have been accustomed to the habitual use of coffee or not? Take for instance typhoid fever—a long siege of suffering—a racked and wrecked nervous system, the chances largely in favor of the patient having been an habitual drinker of coffee, but whether so or not, the coffee is usually not given, though strongly indicated, for the reason that it sustains and supports weary, worn nerves, aids digestion, keeps the alimentary canal, which is alive with germs and putrefactive material, in a more or less antiseptic condition; helps to gently open the sewers of the system, being as it is, a diuretic, a stimulator of the bile flow and other secretions; allays the sense of fatigue and lessens tissue waste; braces up the heart's action and raises arterial tension. We all know that to prevent a chill nothing is superior to a cup of good strong black coffee.

As an antagonist to opium narcosis, strong black coffee stands pre-eminent.

In excess it disorders digestion, removes the desire for food, creates an indisposition to sleep, excites headache, vertigo, mental confusion and disturbing heart action.

